# The Profile and Content of Polyphenolic Compounds and Antioxidant and Anti-Glycation Properties of Root Extracts of Selected Medicinal Herbs

**DOI:** 10.1007/s11130-024-01180-z

**Published:** 2024-04-26

**Authors:** Patrycja Chociej, Kamil Foss, Monika Jabłońska, Małgorzata Ustarbowska, Tomasz Sawicki

**Affiliations:** 1 Department of Human Nutrition, Faculty of Food Sciences, University of Warmia and Mazury in Olsztyn, Słoneczna 45F, Olsztyn, 10-719 Poland; 2 A. Chetnika Vocantional School, Complex No 4, Traugutta 10, Ostrołęka, 07-410 Poland

**Keywords:** Herbs, Flavonoids, Phenolic acids, Antioxidant activity, AGEs inhibition

## Abstract

The objective of our study was to analyse the extracts from six medicinal herb roots (marshmallow, dandelion, liquorice, angelica, burdock, and comfrey) in terms of antioxidant capacity (ABTS, DPPH) and inhibition of advanced glycation end product (AGEs) formation. The quantification of phenolic acids and flavonoids was analysed using the UHPLC-DAD-MS method. Fifteen polyphenolic compounds were detected in the studied herbs. The higher number of polyphenols were found in marshmallows (ten polyphenols), while the lowest was in comfrey (five compounds). Liquorice root revealed the highest individual phenolic concentration (382 µg/g dm) with the higher contribution of kaempferol-3-*O*-rutinoside. Comfrey root extract was characterised by the most abundant TPC (Total Phenolic Content) value (29.79 mg GAE/ g dm). Burdock and comfrey showed the strongest anti-AGE activity studies with the BDA-GLU model. Burdock root was also characterised by the highest anti-AGE activity in the BSA-MGO model. The highest antioxidant capacity was determined by ABTS (72.12 µmol TE/g dw) and DPPH (143.01 µmol TE/g dw) assays for comfrey extract. The *p*-coumaric acid content was significantly correlated with anti-AGE activity determined by the BSA-MGO model. This research sheds new light on the bioactivity of root herbs, explaining the role of *p*-coumaric acid in preventing diabetes.

**Supplementary Information:**

The online version contains supplementary material available at 10.1007/s11130-024-01180-z.

## Introduction

The growing interest in natural remedies and preventive health has drawn the attention of researchers to the therapeutic potential of medicinal herbs, particularly concerning their biological activity [1]. The roots of medicinal herbs, often underexplored compared to their aerial parts, represent a reservoir of bioactive compounds, including polyphenols. Polyphenols play a key role in combating oxidative stress, a key factor linked to the etiology of many chronic diseases. The antioxidant effects of polyphenols are mainly due to their ability to scavenge free radicals, chelate metal ions, and modulate antioxidant defense systems [2]. As a source of polyphenols, herbs can be also potent inhibitors of advanced glycation end-product (AGE) formation. Accumulated high concentrations of AGEs in tissues can contribute to the aging process and the development of various diseases such as Alzheimer’s disease, atherosclerosis, diabetes, kidney disease, and chronic heart failure. The antiglycation properties of polyphenols represent a promising method for the prevention and treatment of these diseases [3]. It is also worth emphasizing that these plant foods are the primary source for most mineral macro- and micro-nutrients, essential elements for human nutrition, and a range of bioactive ingredients, which can also support preventing many chronic diseases [4]. Another thing that should be emphasized is that medical medicinal herbs can be used when preparing meals with a balanced nutritional value and also can play a crucial role in sustainably promoting agricultural production on cultivated lands, facilitating soil carbon sequestration, and taking pressure off global peatlands and forests in terms of a planetary diet [5].

This study aimed to characterize root extracts of selected medicinal herbs, i.e., comfrey, dandelion, licorice burdock and marshmallow roots, in terms of their polyphenolic profile, antioxidant and antiglycation properties. The selection of the roots of specific medicinal herbs for this study is based on their contemporary use in herbal medicine combined with preliminary evidence suggesting a rich polyphenol content. The results of the current study will contribute to a broader understanding of the role of herbal root extracts in health promotion and disease prevention, responding to the growing demand of modern medicine for natural and effective remedies.

## Materials and Methods

The Materials and Methods section is presented as supplementary material.

## Results and Discussion

### Total Phenolic Content

The total phenolic content (TPC) of the examined root herbs ranging from 11.95 to 29.79 mg GAE/g dm (Table 1). Comfrey root exhibited the highest TPC value (29.79 ± 0.41 mg GAE/g dm), while marshmallow root had the lowest (11.95 ± 0.47 mg GAE/g dm). Comfrey root contained 20, 28, 37, 41, and 60% more polyphenols than the angelica, burdock, dandelion, liquorice, and marshmallow root, respectively. TPC values in dandelion and liquorice roots did not differ significantly (*p* > 0.05). Previous studies demonstrated a TPC of 40.51 mg GAE/g for comfrey root [6], while angelica root and burdock root TPC values were similar to those demonstrated previously by the other authors [7, 8]. The literature reported a higher TPC value for dandelion root (178.273 mg GAE/g) [9], while the lowest concentration of polyphenols for liquorice root (6.18 mg GAE/g) [10] and marshmallow root (4.53 mg GAE/g) [11]. The Egyptian ethanolic extract of liquorice was characterized by almost two times higher TPC value than in our study, while the water extract of this same herb had a comparable TPC [12]. Also, a higher TPC value was measured in marshmallow flower water extract, which was 16.56 mg GAE/g dm [13], compared to our study’s 11.95 mg GAE/g dm measurement. Differences in phenolic content may arise from genotype diversity, seasonal and climatic variations and cultivation conditions [14]. Moreover, differences between literature data on total phenolic content may be attributed to extraction method, temperature and physicochemical properties of the solvent used [15].

**Table 1:** Total phenolic content and antioxidant activity determined in root extracts of selected medicinal herbs

Herbs	TPC [mg GAE/g dm]	ABTS [µmol TE/g dm]	DPPH [µmol TE/g dm]
Marshmallow root	11.95 ± 0.47^e^	5.63 ± 0.15^e^	12.64 ± 0.16^e^
Dandelion root	18.91 ± 0.83^d^	8.50 ± 0.53^de^	15.66 ± 0.56^de^
Liquorice root	17.55 ± 0.53^d^	9.39 ± 0.08^cd^	16.60 ± 0.09^d^
Angelica root	23.87 ± 1.08^b^	12.12 ± 1.12^c^	79.76 ± 1.18^c^
Burdock root	21.42 ± 0.87^c^	42.09 ± 1.86^b^	111.35 ± 1.96^b^
Comfrey root	29.79 ± 0.41^a^	72.12 ± 2.10^a^	143.01 ± 2.21^a^

The results are expressed as the mean ± SD (*n* = 3). Different letters depict statistically significant differences (*p* ≤ 0.05) in the same column.

### The Profile of Phenolic Compounds

UHPLC-DAD-MS was used to analyses the profile of polyphenols in the herbal extracts, and the results are presented in Table S2 and Table S3. Fifteen compounds were identified across the studied root herb extracts, encompassing eight phenolic acids (chlorogenic, *m*-hydroxybenzoic, *p*-hydroxybenzoic, caffeic, siringic, gentisic, *p*-coumaric, and ferulic acids) and seven flavonoids ((+)-catechin, quercentin-3-*O*-glucoside, quercentin-3-*O*-vicianoside, kaempferol-3-*O*-rutinoside, kaempferol-3-*O*-glucoside, apigenin and naringenin). Only three compounds were present in all analyzed herbal extracts: *p*-hydroxybenzoic, *p*-coumaric, and ferulic acids. Quercetin-3-*O*-glucoside was found in five of six examined samples (excluding comfrey root). Syringic acid was present in five herb root extracts (marshmallow, dandelion, liquorice, burdock, and comfrey root) with a trace contribution. (+)-Catechin was present in four samples (marshmallow, liquorice, angelica, and comfrey root). Gentisic acid and apigenin were found only in burdock root. Caffeic acid, quercetin-3-*O*-vicianoside, and naringenin were decocted only in angelica root, liquorice root, and marshmallow root, respectively. The dominant compound in marshmallow, dandelion, and burdock was quercetin-3-*O*-glucoside, with the highest contribution of 40.5% in dandelion root extracts. Caffeic acid was the main compound in angelica root extracts, while ferulic acid was in comfrey root. Liquorice root extracts were characterized by the higher contribution of kampferol-3-*O*-rutinoside (25.8% of the total phenolic index -TPI).

Nastić et al. [16] found eleven phenolic compounds in comfrey root from Serbia; eight compounds belong to the phenolic acids and three to flavonoids. In the cited study, the comfrey root did not contain four phenolics (p-hydroxybenzoic acid, syringic acid, catechin, and quercetin-3-*O*-glucoside), which were noted in our study. On the other hand, Nastić et al. [16] detected gallic acid, protocatechuic acid, β-resorcylic acid, sinapic acid, naringin, rutin, cinnamic acid, and naringenin. The same authors [16] showed that *p*-coumaric acid was a predominant compound with the contribution of 24% of the polyphenols detected in the comfrey extract; while in the current study ferulic acid was the most abundant compound A small number of polyphenols (rosmarinic acid, globoidnan A, globoidnan B, rabdosiin) were found in comfrey root collected from different European regions [17]. In the case of dandelion root, the Ukrainian herb sample was analyzed with only five phenolic compounds (chlorogenic acid, caffeic acid, rutin, luteolin, and ferulic acid) [18]. The main compounds were chlorogenic acid (1340 µg/g), followed by luteolin (1080 µg/g). In comparison, in Mongolia’s dandelion extracts seven flavonoids were identified (hesperetin-5′-*O*-β-rhamnoglucoside, quercetin, hesperetin-7-glucuronide, kaempferol-3-glucoside, baicalein, hyperseroside, and rutin) [19]. On the other hand, in the root and herb juice of dandelion, Schütz et al. [20] detected forty-three polyphenols, including mono- and caffeoylquinic acids, tartaric acid derivatives, flavone and flavonol glycosides. The methanol/water extracts of burdock root from Iran contained only four phenolic compounds (chlorogenic acid, trans-ferulic acid, hesperidin, and rosmarinic acid) [21], contrary in our study we detected two times more compounds. In Irania’s burdock root sample, the predominant compound was hesperidin, which was not detected in the present study. In comparison, the study conducted by Ferracane et al. [22] detected one more phenolic in burdock roots. The cited research noted chlorogenic acid, dicaffeoylqunic acid, quercetin rhamnosied, arctiin, quercetin, and luteolin. Results of the study conducted by Farhat et al. [13] showed the presence of five phenolic acids (syringic, gallic, caffeic, *p*-coumaric and *trans*-ferulic acids) and eight flavonoids (catechin, apigenin, chrysin, quercetin, kaempferol, genistein, rutin trihydrate and galangin) in the marshmallow flower water extract. The predominant compound was caffeic acid (0.302 µg/g dm); while in our study we did not detect caffeic acid in the marshmallow root extract. In the case of liquorice root, Egyptian ethanol extract contains thirty-nine phenolic compounds, with a higher level of pyrogallol [12]. Montoro et al. [23] showed thirteen flavonoids in liquorice root were derived from China, Iran, Turkey, and Italy. Moreover, these authors detected liquiritin and isoliquiritin, which are widely known as active compounds of these plant materials. Also, the presence of these compounds in liquorice roots was confirmed in studies conducted by Farag et al. [24]. In our research, we did not identify these compounds due to the lack of standards to establish optimal conditions in a targeted analysis of analysed groups of substances. In the angelica root Kozłowska et al. [25] found only five phenolics in the aqueous/ethanolic extracts, including four phenolic acids (chlorogenic acid, ferulic acid, cichoric acid, and isochlorogenic acid B) and one flavonoid (tiliroside). Only ferulic acid was detected in our and Kozłowska et al. [25] studies. In contrast, these authors examined a nearly three times higher ferulic acid content (200 µg/g) as a measurement of our research. In the cited study, the predominant compound was chlorogenic acid (58%).

In the current study, the highest total phenolic index (TPI) was measured in liquorice root (382.10 µg/g), followed by angelica root (320.02 µg/g). Liquorice root extracts showed a more than twofold higher TPI than burdock, dandelion, and comfrey (Table S2). This can be attributed to the fact that the extracts of liquorice root were characterized by one of the highest numbers of detected phenolic compounds (one less than marshmallow root) and a high concentration of four compounds with more than 15% contribution in TPI.

The obtained results showed that certain compounds were present in all analyzed extracts, whereas others were unique to a specific medicinal plant. As mentioned above, the chemical composition of herbs can be influenced by several factors, including environmental growth factors such as temperature, soil and light, as well as the timing of harvesting or storage conditions [14, 26] and method of extraction [15].

### Antioxidant Capacity

In the current study, the antioxidant capacities of various herbal root extracts were evaluated using ABTS and DPPH assays, focusing on their free radical-scavenging abilities. Comfrey root emerged as the most potent antioxidant among the examined samples, exhibiting ABTS and DPPH values of 143.01 µmol TE/g and 72.12 µmol TE/g, respectively (Table 1). Burdock root demonstrated notable antioxidant activity, evident in the ABTS assay (42.09 µmol TE/g) and the DPPH assay (111.35 µmol TE/g). Angelica root exhibited moderate antioxidant values (ABTS: 79.76 µmol TE/g, DPPH: 12.12 µmol TE/g). In contrast, liquorice, dandelion and marshmallow roots demonstrated lower antioxidant activity in the ABTS and DPPH assays compared to the values obtained for burdock and comfrey roots (*p* < 0.05; Table 1) with values of 16.60 µmol TE/g and 9.39 µmol TE/g for liquorice, 15.66 µmol TE/g and 8.50 µmol TE/g) for dandelion and 12.64 µmol TE/g and 5.63 µmol TE/g for marshmallow, respectively. Studies by other researchers on the herbs in question have consistently confirmed their antioxidant properties [13, 27–29]. In the current study high ABTS and DPPH scores are strongly associated with high TPC values, which means that phenolic compounds contribute greatly to their antioxidant properties [30]. This conclusion is consistent with the literature, which confirms the significant effect of polyphenols on antioxidant activity [30, 31].

### Inhibition of AGEs Formation

**Fig. 1:**
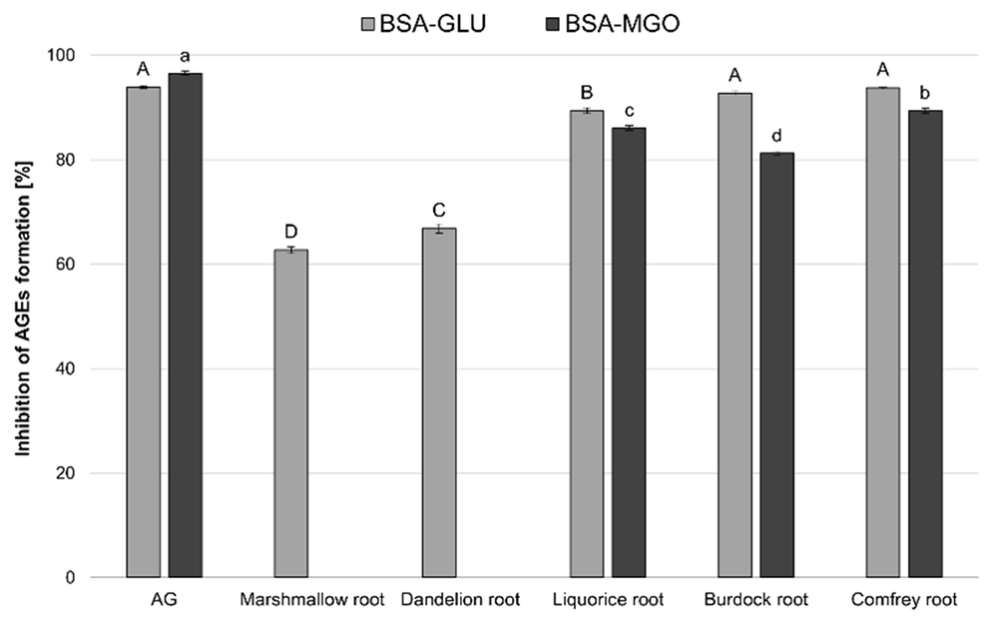
The inhibitory effects of root herbs against advanced glycation end-products (AGEs) formation. The results are expressed as the mean ± SD (*n* = 3). Different letters indicate significant differences (*p* < 0.05). AG – aminoguanidine as a positive control; BSA – bovine serum albumin with glucose (GLU) or methylglyoxal (MGO) assays

Examination of different herbal root extracts for their ability to inhibit AGEs formation has provided valuable insights into their potential health benefits. The results of the BSA-GLU assay demonstrated that the analyzed herbal root extracts exhibited distinct inhibitory effects on the formation of AGEs. Among the examined samples, burdock and comfrey demonstrated the highest inhibitory effects, closely mirroring the control group with approximately 93% inhibition (Fig. 1). Liquorice also exhibited significant inhibition (89.4%), although it did not reach the level of the control. Marshmallow and dandelion roots showed moderate inhibitory effects, ranging from 62.7 to 66.8% of inhibition. Consistent with the outcomes observed in the BSA-GLU assay, the results of the BSA-MGO assay demonstrated that comfrey, burdock and liquorice roots exhibited inhibitory effects on AGEs formation (ranging from 81.2 to 89.4%). The observed substantial inhibitory effects of these herbs in both the BSA-GLU and BSA-MGO assays may indicate their potential in inhibiting glycation processes across various pathways linked to not just high blood sugar but also oxidative stress. In contrast, dandelion and marshmallow roots showed no inhibitory effects on AGEs formation in the BSA-MGO assay suggesting a limited impact on glycation processes involving methylglyoxal. On the other hand, extracts with selective inhibitory effects (dandelion, marshmallow) may provide valuable insights into the specific mechanisms through which they exert their anti-glycation properties. Interestingly, angelica root, evaluated in both the BSA-GLU and BSA-MGO assays, did not exhibit inhibitory effects on AGEs formation in either method possibly due to its to its specific bioactive substance composition. Integration of the results from glycation assays with TPC data revealed that the observed anti-glycation properties of the analyzed herbs may be attributed, at least in part, to their phenolic content. It should be noted, however, that other bioactive compounds may also play a role in the observed effects, and further studies are needed to identify specific components responsible for the anti-glycation activity in these plant roots.

Our results align with those of Siddiqui et al. [32] and Khan et al. [33], who demonstrated that root extracts of liquorice and burdock effectively inhibited the formation of AGEs, respectively. To the best of our knowledge, no reports in the literature describe the in vitro anti-glycation activity of the other roots analyzed in the current study (marshmallow, dandelion, comfrey root).

### Correlation Study and Principal Component Analysis (PCA)

**Fig. 2:**
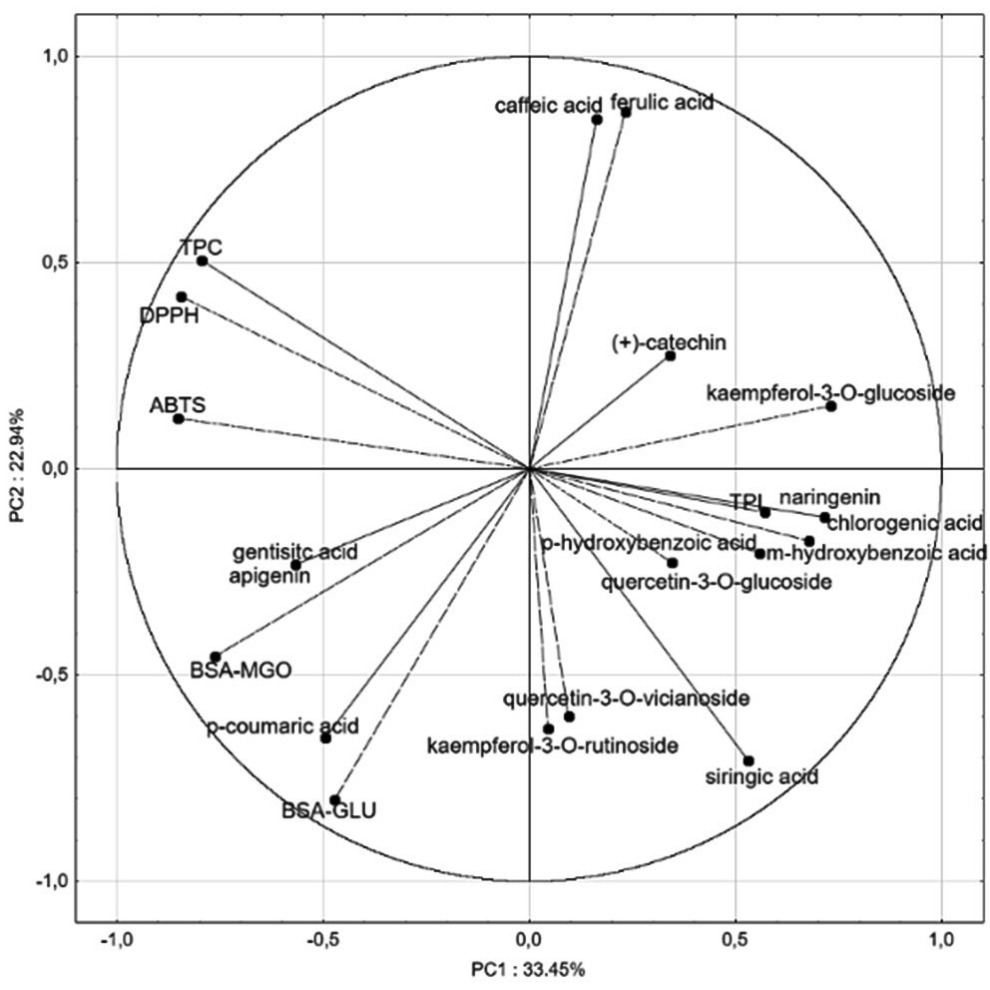
Principal components plot of the total content and qualitative composition of polyphenols as well as antioxidant capacity (ABTS, DPPH) and inhibitory effects against advanced glycation end-products formation variables for the root herbs extracts against principal component 1 (PC1) and principal component 2 (PC2).

Principal component analysis (PCA) showed a correlation between the principal components and the original variables in the coordinate system of the first two principal components (Fig. 2), PC1 (33.45%) and PC2 (22.94%). PCA revealed that parameters such as TPC, DPPH, and ABTS were located close to one another in the PCA plot due to the presence of strong and significant correlations. Moreover, the Pearson’s correlation analysis (Table S4) showed a high and statistical significantly correlation between DPPH and ABTS (*r* = 0.909, *p* < 0.05), BSA-MGO model system and *p*-coumaric acid (*r* = 0.880, *p* < 0.05), and between TPC and DPPH (*r* = 0.871, *p* < 0.05). Positive correlation was obtained between TPC and ABTS (*r* = 0.802). Medium positive correlation was observed between BSA-GLU and BSA-MGO model systems (*r* = 0.747), BSA-MGO model system and ABTS (*r* = 0.680), and between BSA-GLU model system and *p*-coumaric acid (*r* = 0.649). The obtained data suggested that TPC are mostly responsible for the antioxidant properties of root herb extracts, whereas the presence of *p*-coumaric acid is responsible for the glycation inhibitory activity. The obtained results are consistent with those presented by Ulewicz-Magulska and Wesołowski [31], who found that TPC significantly contributed to the antioxidant capacity of herb extracts. Our results are agreement with Starowicz and Zieliński [3], who also showed high correlation coefficient between BSA-MGO and ABTS in different herb extracts. Additionally, our results align with previous studies that demonstrated that *p*-coumaric acid effectively inhibited the formation of AGEs [34, 35]. In the current study no significant correlation were found between TPC and anti-AGEs activity, aligning with findings in kiwifruit extracts [35]. It is well known that the polyphenol profile and content of herbs is strongly influenced by many factors (vegetation season, climatic and cultivation conditions), and also by sample preparation and analytical methods [30].

## Conclusions

Considering the obtained data, we may conclude that analysed marshmallow, dandelion, liquorice, angelica, burdock, and comfrey root herbs are important sources of polyphenolic compounds, particularly *p*-coumaric acid, ferulic acid, and quercetin-3-*O*-glucoside, the content of which is crucial for bioactivity properties. Understanding their health-promoting potential and incorporating them into daily consumption could enhance nutritional strategies in preventing lifestyle diseases, particularly type 2 diabetes.

### Electronic Supplementary Material

Below is the link to the electronic supplementary material.

Supplementary Material 1

## Data Availability

No datasets were generated or analysed during the current study.
