# Supplementary material for: The Profile and Content of Polyphenolic Compounds and Antioxidant and Anti-Glycation Properties of Root Extracts of Selected Medicinal Herbs
Source: Plant Foods Hum Nutr. 2024 Apr 26;79(2):468–73. doi: 10.1007/s11130-024-01180-z (PMC11178571; doi:10.1007/s11130-024-01180-z)
Supplement: Supplementary file 1 — Supplementary Material 1 [file 11130_2024_1180_MOESM1_ESM.pdf]

Supplementary material

The profile and content of polyphenolic compounds and antioxidant and anti-glycation properties of root extracts of selected medicinal herbs

Patrycja Chociej[1], Kamil Foss[1], Monika Jabłońska[1]*, Małgorzata Ustarbowska[2], Tomasz Sawicki[1]*

[1] Department of Human Nutrition, Faculty of Food Sciences, University of Warmia and Mazury in Olsztyn, Słoneczna 45F, 10-719 Olsztyn, Poland

[2] A.Chetnika Vocantional School Complex No 4, Traugutta 10, 07-410 Ostrołęka, Poland

* Corresponding author: Tomasz Sawicki, tomasz.sawicki@uwm.edu.pl; Monika Jabłońska, monika.jablonska@uwm.edu.pl

Materials and Methods

Chemicals

The following chemicals and reagents were purchased from Sigma Chemical Co. (St. Louis, MO, USA): Folin phenol reagent, 6-hydroxyl-2,5,7,8-tetramethyl-chroman-2-carboxylic acid (Trolox), 2,2′-azinobis(3-ethylbenzothiazoline-6-sulphonic acid) diammonium salt (ABTS), 2,2-diphenyl-1-picrylhydrazyl (DPPH), aluminium chloride ($AlCl_3$), sodium nitrite ($NaNO_2$), glucose (GLU), methylglyoxal (MGO), bovine serum albumin (BSA), aminoguanidine hydrochloride, and HPLC-grade solvents (water, acetonitrile, methanol and formic acid). Standards of phenolic acids (benzoic, gallic, caffeic, chlorogenic, ellagic, ferulic, *p*-coumaric, *t*-cinnamic, *m*-hydrocxybenzoic, *p*-hydrocxybenzoic, rosmarinic, syringic, gentisic and vanillic acids), and flavonoids (quercetin, kaempferol, naringenin, rutin, apigenin, (+)-catechin, and quercetin-3-*O*-glucoside, kaempferol-3-*O*-glucoside) were purchased from Sigma Chemical Co. (St. Louis, MO, USA).

Material

The six herbs used in this study were purchased in a specialized herbal store in Olsztyn, Poland market in 2023. The herbs included marshmallow root (*Althaea officinalis* L.), dandelion root (*Taraxacum officinale* F.H. Wigg.), liquorice root (*Glycyrrhiza glabra* L.), burdock root (*Arctium lappa* L.), comfrey root (*Symphytum officinale* L.), and angelica root (*Angelica archangelica* L.).

Extraction procedure

Powdered herb samples (200 mg) were extracted with 1 mL of methanol 80% (v/v), vortexed for 30 s, and placed in the ultrasonic bath for 30 s. The vortex and ultrasound

application were repeated three times. After that, the samples were centrifugated at 14,000 rpm (VWR micro star 30R, Radnor, PA, USA) for 10 min at 4 °C. The obtained supernatants were transferred to 5 mL vials and the herb residues were re-suspended in fresh extraction solvent. These steps were repeated 5 times until 5 mL of each herb supernatant had been extracted. Each herb sample was prepared in three replications.

The above procedure was carried out to obtain material for determining the antioxidant capacity (AC), total polyphenol content (TPC), anti-AGEs potential and profile of polyphenols. However, extracts to analyse the antiglycaemic activity, the solvent was evaporated under nitrogen, and the obtained material was dissolved in a suitable solvent used in the abovementioned test.

The analysis was performed in triplicate.

Total phenolic content (TPC)

The total phenolic content (TPC) was carried out using the Folin-Ciocalteu reagent according to the procedure described by Foss et al. [1]. A mixture containing 15 µL of appropriately diluted extract fractions and 240 µL of Folin's phenol reagent were placed into wells of microplates and incubated for 10 min at room temperature (RT). Next, 15 µL of 20% sodium carbonate was added and shaken. Absorbance was measured at 765 nm using a microplates reader (FLUOstar Omega, BMG LABTECH, Ortenberg, Germany). Obtained result were expressed as mg gallic acid equivalents (GAE) per gram of sample. All measurements were performed in triplicates.

Determination of polyphenols profile by UHPLC-DAD-MS

The analysis of polyphenols was performed according to the methodology described by Sawicki et al. [2]. Polyphenols qualitative and quantitative were carried out using a UHPLC

system (Nexera XR, Shimadzu, Japan) coupled with a diode area detector (DAD) and mass spectrometer (LCMS-2020, Shimadzu, Japan). Measurement parameters were as follows: eluent 0.01% formic acid in water with 2 mM ammonium formate (A) and 0.01% formic acid in 95% acetonitrile solution with 2 mM ammonium formate (B); flow rate 0.15 mL/min; scanning in negative ionization; column Column C18 BEH (1.7 µm particle size; 100 × 2.1 mm; Waters, Warsaw, Poland); oven temperature was 50 °C; sample injection volume 10 µL. An analysis was conducted in the selected ion monitoring mode (SIM). Analysed compounds were identified based on their qualitative ions, retention times and λmax value with the previously published data [2, 3]. The quantity of polyphenols was calculated from the UHPLC-DAD-MS peak area against commercially available standards. The UHPLC-DAD-MS data of herb phenolic acids and flavonoids are presented in Table S1.

Antioxidant capacity

ABTS assay

The measurement was carried out according to the method described by Horszwald and Andlauer [4]. Briefly, ABTS•+ solution was diluted with a water:methanol mixture (20:80, v/v) to an absorbance level of 0.70 ± 0.02 at 734 nm. For the spectrophotometric assay, 290 µL of the ABTS•+ solution and 10 µL of the respective extract, Trolox or blank (80% methanol) were mixed, and absorbance was measured at 734 nm directly after 6 min incubation at 30 °C using a microplate reader (FLUOstar Omega, BMG LABTECH, Ortenberg, Germany). The response of the calibration curve was linear from 0.01 to 2.0 mM (R2 = 0.999). The results were expressed as µmol of Trolox equivalents (TE) /g of sample. Analysis was carried out in triplicate.

DPPH assay

Determination of antioxidant properties by the DPPH• method was conducted according to the procedure developed by Horszwald and Andlauer [4]. The DPPH• radical solution was prepared by dissolving 10 mg of DPPH• in 250 mL of 80% methanol. To perform the spectrophotometric test, 300 μL of DPPH• solution and 20 μL of appropriately diluted sample or Trolox solution were mixed. The resulting mixture was left for 30 min at room temperature in the dark. Decreasing absorbance of the resulting solution was monitored at 517 nm using a microplate reader FLUOstar Omega (BMG LABTECH, Ortenberg, Germany). The standard curve was plotted based on the length of the lag phase versus Trolox concentrations within the range of 0.01 - 2.0 mM (R^2 = 0.998). The results were expressed as μmol of TE/g of sample. Analysis was carried out in triplicate.

In vitro antiglycation activity

BSA-GLU assay

Inhibition of AGEs formation in the BSA-GLU test was assessed following the method of Przygodzka and Zieliński [5]. One millilitre of a mixture of *D*-glucose (1.0 M), BSA (10 mg/mL) and sodium azide (0.1 mg/mL) in phosphate buffer (0.1 M, pH 7.4) was incubated (55 °C for 3 days) with or without 1 mL of the analyzed extract dissolved in phosphate buffer. Three hundred microliters of the obtained material was placed into 96-well plates, and the formation of AGEs was determined based on the measurements of fluorescence at λ = 330 nm (excitation wave) and at λ = 410 nm (emission wave) (FLUOstar Omega, BMG LABTECH, Ortenberg, Germany). The positive control was aminoguanidine. The analysis was carried out in triplicate. The mathematical formula was used to calculate the percent inhibition of AGEs formation by the tested extracts (Formula 1).

% inhibition = (1- (fluorescence of the sample/ fluorescence of the blank)) * 100% (1)

BSA-MGO assay

Inhibition of AGEs formation in the BSA-MGO test was assessed following the method of Przygodzka and Zieliński [5]. 1 mL of a mixture of BSA (1 mg/mL), MGO (5 mM) and sodium azide (0.1 mg/mL) in phosphate buffer (0.1 M, pH 7.4) was incubated (37 °C for 7 days) with or without 1 mL of the tested extracts dissolved in phosphate buffer. Three hundred microliters of the obtained material was placed into 96-well plates, and the formation of AGEs was determined based on the measurements of fluorescence at $\lambda = 340$ nm (excitation wave) and at $\lambda = 420$ nm (emission wave) (FLUOstar Omega, BMG LABTECH, Ortenberg, Germany). The positive control was aminoguanidine. The analysis was carried out in triplicate.

Statistical analysis

The data are presented as mean values ± standard deviations of triplicate measurement. The differences between samples were analyzed by a one-way ANOVA with Tukey's test ($p < 0.05$). Pearson's linear correlation was applied to measure the statistical relationship between anti-AGEs, ABTS, DPPH parameters, individual phenolics, and TPC. Principal component analysis (PCA) was performed based on a correlation matrix to reduce the data's dimensionality and present the samples in a new coordinate system. The statistical analysis was performed using STATISTICA 13.0 (StatSoft Inc., Tulsa, OK, USA).

References

1. Foss K, Przybyłowicz KE, Sawicki T (2022) Antioxidant Activity and Profile of Phenolic Compounds in Selected Herbal Plants. Plant Foods Hum Nutr 77(3):383-389. https://doi.org/10.1007/s11130-022-00989-w
2. Sawicki T, Błaszczak W, Latocha P (2023) In vitro anticholinergic and antiglycaemic properties of frost-hardy Actinidia fruit extracts and their polyphenol profile, L-ascorbic acid content and antioxidant capacity. Food Res Int 173:1–8. https://doi.org/10.1016/j.foodres.2023.113324

3. Zakrzewski A, Purkiewicz A, Jakuć P, Wiśniewski P, Sawicki T, Chajęcka- Wierzchowska W, Tańska M (2022) Effectiveness of various solvent-produced thyme (Thymus vulgaris) extracts in inhibiting the growth of Listeria monocytogenes in frozen vegetables. NFS Journal 29:26–34. https://doi.org/10.1016/j. nfs.2022.09.004
4. Horszwald A, Andlauer W (2011) Characterisation of bioactive compounds in berry juices by traditional photometric and modern microplate methods. J Berry Res 1:189–199. https://doi.org/10.3233/JBR-2011-020
5. Przygodzka M, Zieliński H (2015) Evaluation of in vitro inhibitory activity of rye-buckwheat ginger cakes with rutin on the formation of advanced glycation end-products (AGEs). Pol J Food Nutr Sci 65:191‑198. https://doi.org/10.1515/pjfns-2015-0038

Table S1. The parameter of the analyzed phenolic acids and flavonoids

Compounds	R_t [min]	$[M]^-$ (m/z)	λ_{max} [nm]
Phenolic acids			
gallic acid	2.71	169	272
gentisic acid	3.31	153	256
rosmarinic acid	4.49	359	328
chlorogenic acid	5.15	353	298
caffeic acid	5.84	179	323
syringic acid	5.88	197	320
vanillic acid	6.01	167	260/292
ellagic acid	6.64	301	265
p-coumaric acid	6.72	163	309
ferulic acid	7.17	193	322
m-hydroxybenzoic acid	7.00	137	255
benzoic acid	8.13	121	228/272
p-hydroxybenzoic acid	8.77	137	255
t-cinnamic acid	9.21	147	307
Flavonoids			
(+)-catechin	5.38	289	242
rutin	5.96	609	257/355
kaempferol	6.04	285	345
quercetin-3-*O*-glucoside	6.81	463	254/355
kaempferol-3-*O*-glucoside	7.03	447	265/352
quercetin	8.83	301	255/350
apigenin	9.32	269	267/336
naringenin	9.75	271	288

Abbreviations: R_t – retention time; $[M]^-$ (m/z) – parent ion; λ_{max} [nm] – absorption maxima

Table S2. Total phenolic index (TPI) and contribution of individual phenolic compounds in root extracts of selected medicinal herbs

Phenolic compounds	% of contribution in TPI					
	Root herbs					
	Marshmallow root	Dandelion root	Liquorice root	Angelica root	Burdock root	Comfrey root
chlorogenic acid	0.3	0.0	0.0	0.0	0.0	0.0
m-hydroxybenzoic acid	0.4	0.6	0.0	0.0	0.0	0.0
p-hydroxybenzoic acid	1.1	2.3	0.4	0.3	0.6	2.0
caffeic acid	0.0	0.0	0.0	34.0	0.0	0.0
siringic acid	1.0	0.8	0.5	0.0	0.5	0.8
gentisic acid	0.0	0.0	0.0	0.0	0.2	0.0
p-coumaric acid	12.6	19.8	9.3	11.0	18.9	26.6
ferulic acid	23.6	36.1	16.9	21.1	34.5	49.5
(+)-catechin	10.3	0.0	7.3	8.9	0.0	21.1
quercetin-3-*O*-glucoside	25.7	40.5	18.9	22.5	38.2	0.0
quercetin-3-*O*-vicianoside	0.0	0.0	19.1	0.0	0.0	0.0
kaempferol-3-*O*-rutinoside	0.0	0.0	25.8	0.0	4.7	0.0
kaempferol-3-*O*-glucoside	2.4	0.0	1.7	2.2	0.0	0.0
apigenin	0.0	0.0	0.0	0.0	2.4	0.0
naringenin	22.5	0.0	0.0	0.0	0.0	0.0
**TPI***	**278.53±0.52^{c}**	**177.80±0.09^{e}**	**382.10±1.86^{a}**	**320.02±016^{b}**	**187.21±1.09^{d}**	**132.73±0.33^{f}**

The results are expressed as the mean ± SD. Different letters depict statistically significant differences ($p \leq 0.05$). *Total phenolic index (TPI) calculated by the sum of individual phenolics identified in the tested extracts. Values were expressed as micrograms per gram of sample (μg/g).

Table S3. The content of polyphenols (μg/g sample) detected in the selected medicinal medicinal herbs

Phenolic compounds	Root herbs					
	Marshmallow root	Dandelion root	Liquorice root	Angelica root	Burdock root	Comfrey root
chlorogenic acid	0.93±0.04	nd	nd	nd	nd	nd
m-hydroxybenzoic acid	1.07	1.10	nd	nd	nd	nd
p-hydroxybenzoic acid	3.10±0.05[b]	4.02±0.06[a]	1.72±0.07[d]	1.01±0.02[e]	1.12±0.05[e]	2.71±0.14[c]
caffeic acid	nd	nd	nd	108.91±0.02	nd	nd
siringic acid	2.90±0.21[a]	1.36±0.03[c]	2.02±0.14[b]	nd	1.02±0.06[c]	1.11±0.06[c]
gentisic acid	nd	nd	nd	nd	0.41±0.01	nd
p-coumaric acid	35.17±0.00[c]	35.17±0.00[c]	35.38±0.03[a]	35.17±0.00[c]	35.32±0.00[b]	35.25±0.01[c]
ferulic acid	65.81±0.63[b]	64.16±0.06[c]	64.52±0.13[c]	67.40±0.02[a]	64.51±0.02[c]	65.68±0.16[b]
(+)-catechin	28.57±0.05[a]	nd	27.95±0.00[b]	28.56±0.14[a]	nd	27.98±0.04[b]
quercetin-3-*O*-glucoside	71.59±0.00[c]	72.00±0.00[b]	72.30±0.06[a]	71.93±0.01[b]	71.48±0.01[d]	nd
quercetin-3-*O*-vicianoside	nd	nd	73.12±0.03	nd	nd	nd
kaempferol-3-*O*-rutinoside	nd	nd	98.76±2.81[a]	nd	8.88±0.43[b]	nd
kaempferol-3-*O*-glucoside	6.82±0.06[a]	nd	6.33±0.25[b]	7.05±0.03[a]	nd	nd
apigenin	nd	nd	nd	nd	4.47±0.00	nd
naringenin	62.57±0.01	nd	nd	nd	nd	nd
**TPI***	**278.53±0.52[c]**	**177.80±0.09[e]**	**382.10±1.86[a]**	**320.02±016[b]**	**187.21±1.09[d]**	**132.73±0.33[f]**

The results are expressed as the mean ± SD. Different letters depict statistically significant differences ($p \leq 0.05$). *Total phenolic index (TPI) calculated by the sum of individual phenolics identified in the tested extracts. Values were expressed as micrograms per gram of sample (μg/g).

Table S4. Pearson's correlation coefficients denoting the relationships between the content of total polyphenols (TPC), individual identified phenolics, TPI, and antioxidant capacity (DPPH, ABTS), and anti-AGEs (BSA-GLU, BSA-MGO) activity

	**BSA-GLU**	**BSA-MGO**	**DPPH**	**ABTS**
chlorogenic acid	-0.067	-0.447	-0.438	-0.355
gentisitc acid	0.345	0.401	0.418	0.314
p-coumaric acid	0.649	0.880*	0.151	0.257
syringic acid	0.476	-0.018	-0.604	-0.308
caffeic acid	-0.926*	-0.447	0.144	-0.236
m-hydroxybenzoic acid	-0.060	-0.706	-0.672	-0.518
p-hydroxybenzoic acid	0.236	-0.370	-0.417	-0.120
ferulic acid	-0.787	-0.383	0.270	-0.008
(+)-catechin	-0.278	0.020	-0.009	-0.019
naringenin	-0.067	-0.447	-0.438	-0.355
apigenin	0.345	0.401	0.418	0.314
quercetin-3-O-glucoside	-0.360	-0.486	-0.696	-0.867*
quercetin-3-O-vicianoside	0.299	0.452	-0.404	-0.286
kaempferol-3-O-rutinoside	0.335	0.495	-0.372	-0.261
kaempferol-3-O-glucoside	-0.562	-0.368	-0.501	-0.651
TPI	-0.384	-0.146	-0.568	-0.697
TPC	0.016	0.444	0.871*	0.802
BSA-GLU	1.000	0.747	0.143	0.482
BSA-MGO		1.000	0.531	0.680
DPPH			1.000	0.909*
ABTS				1.000

* correlation is significant when $p \leq 0.05$
